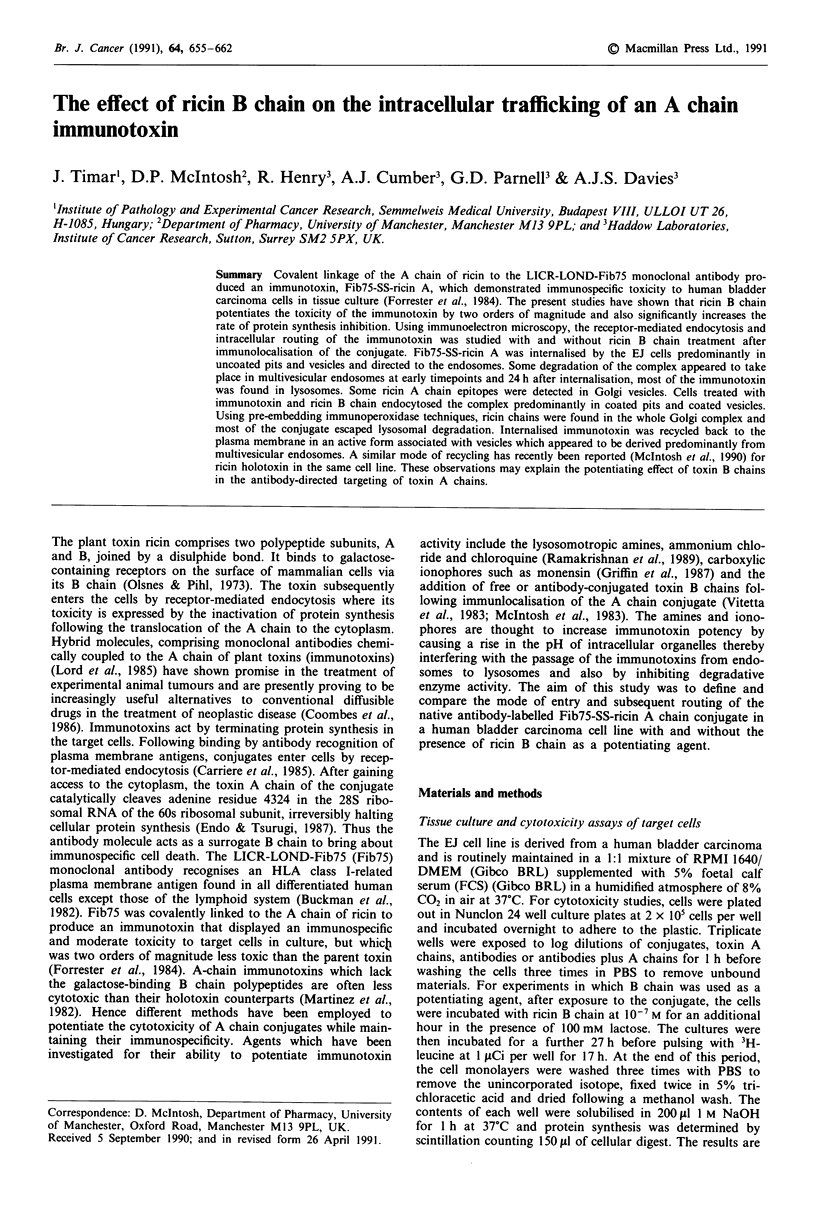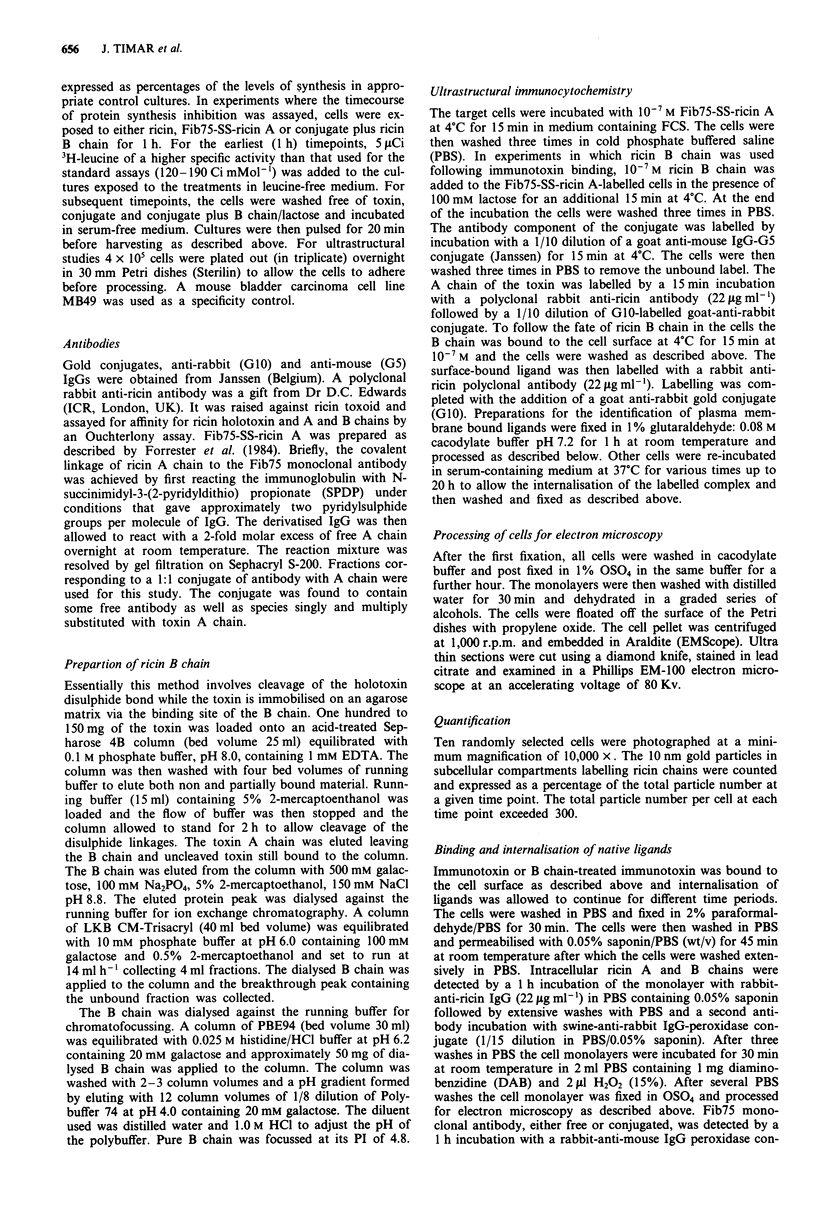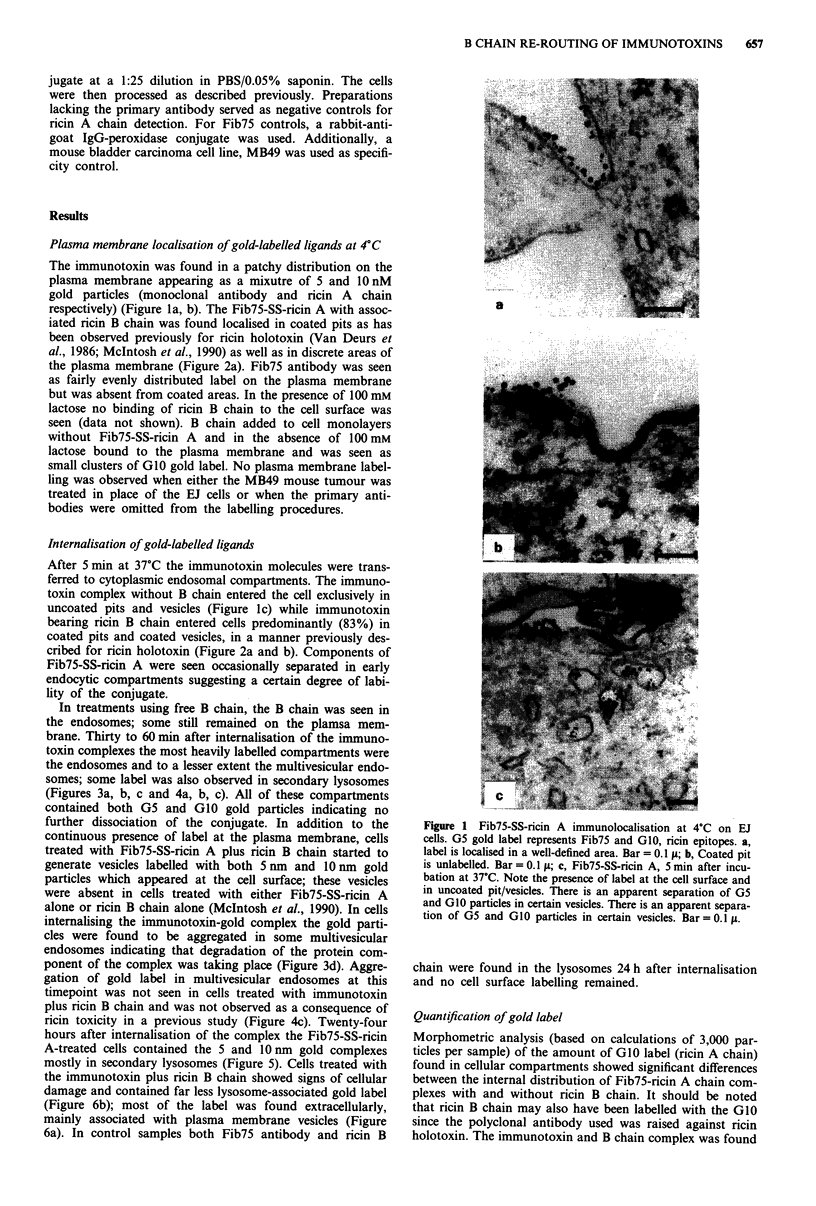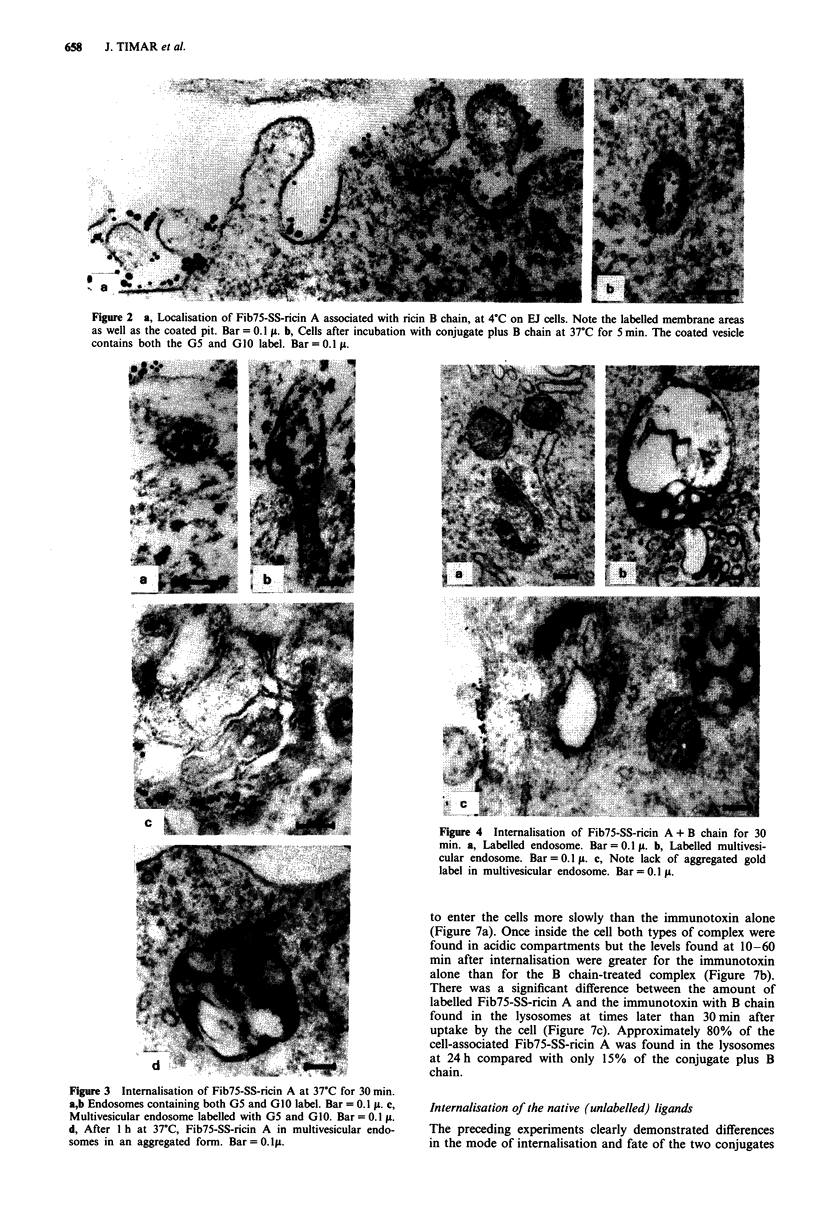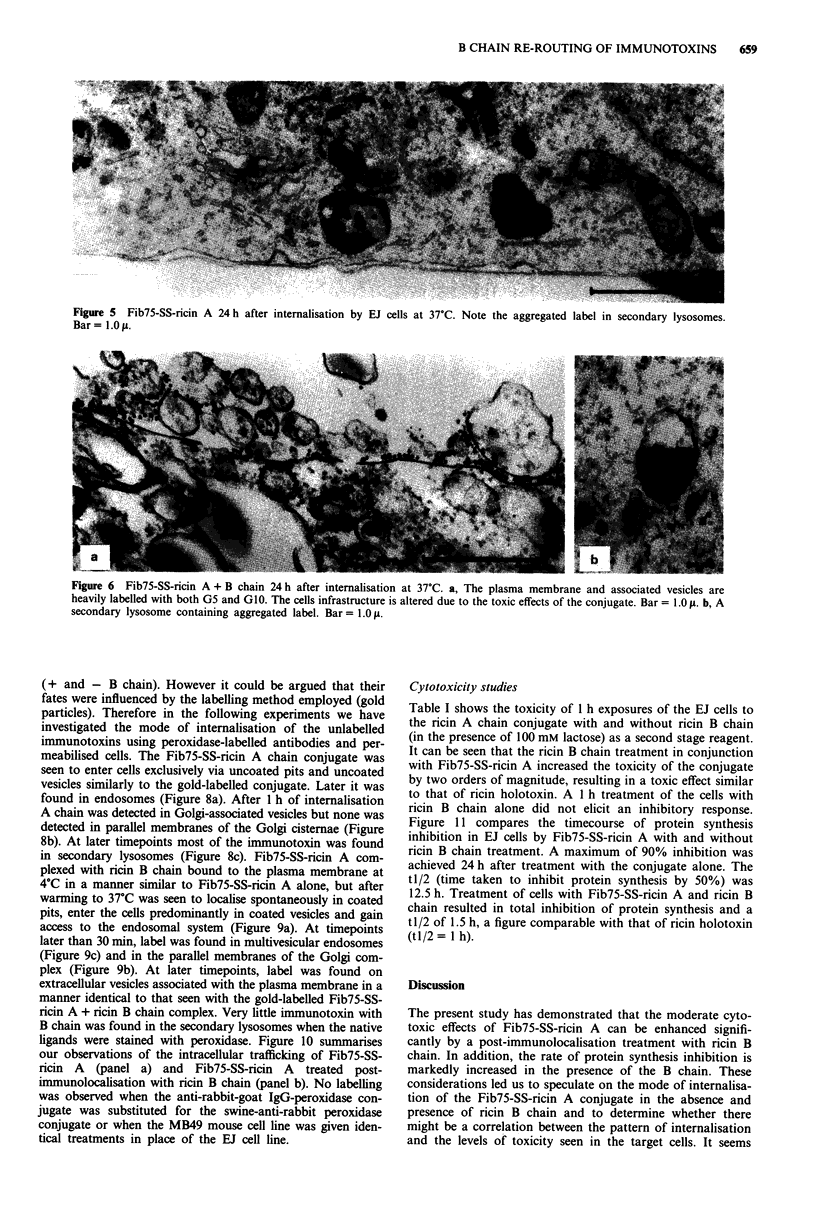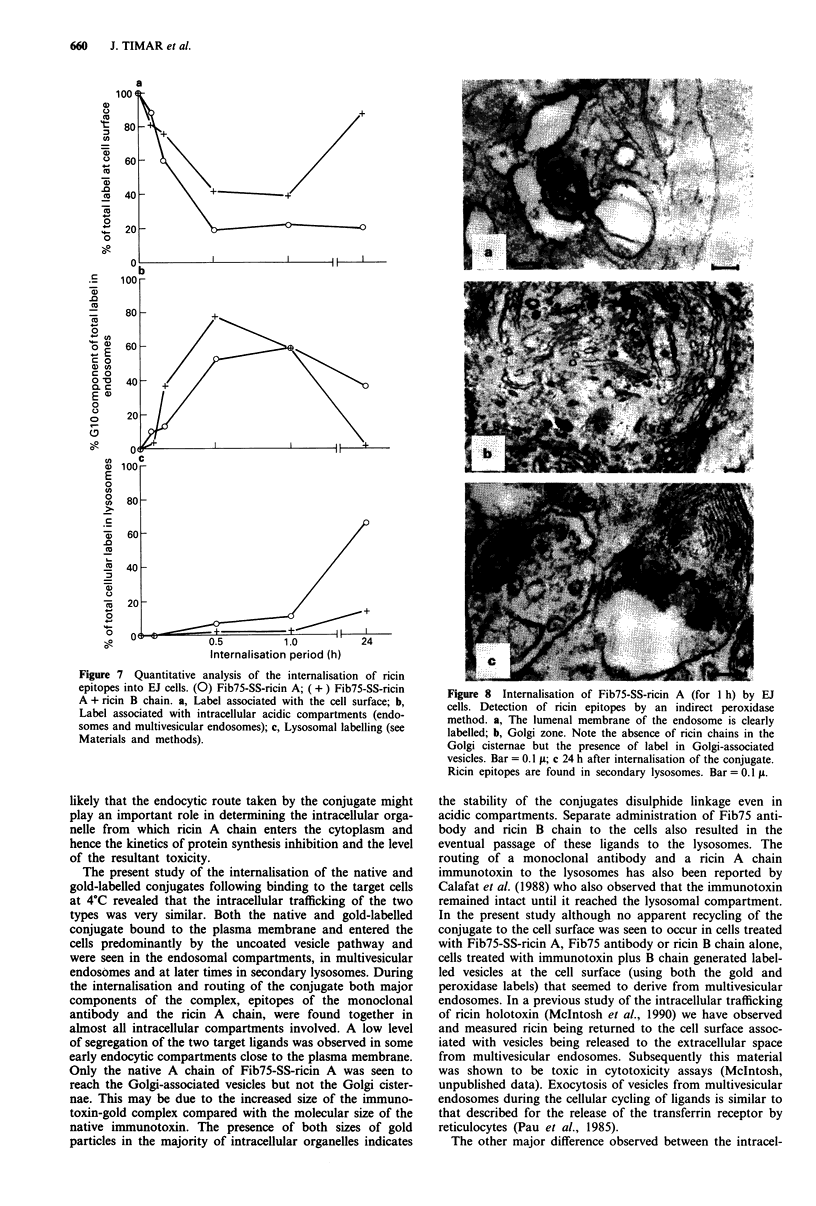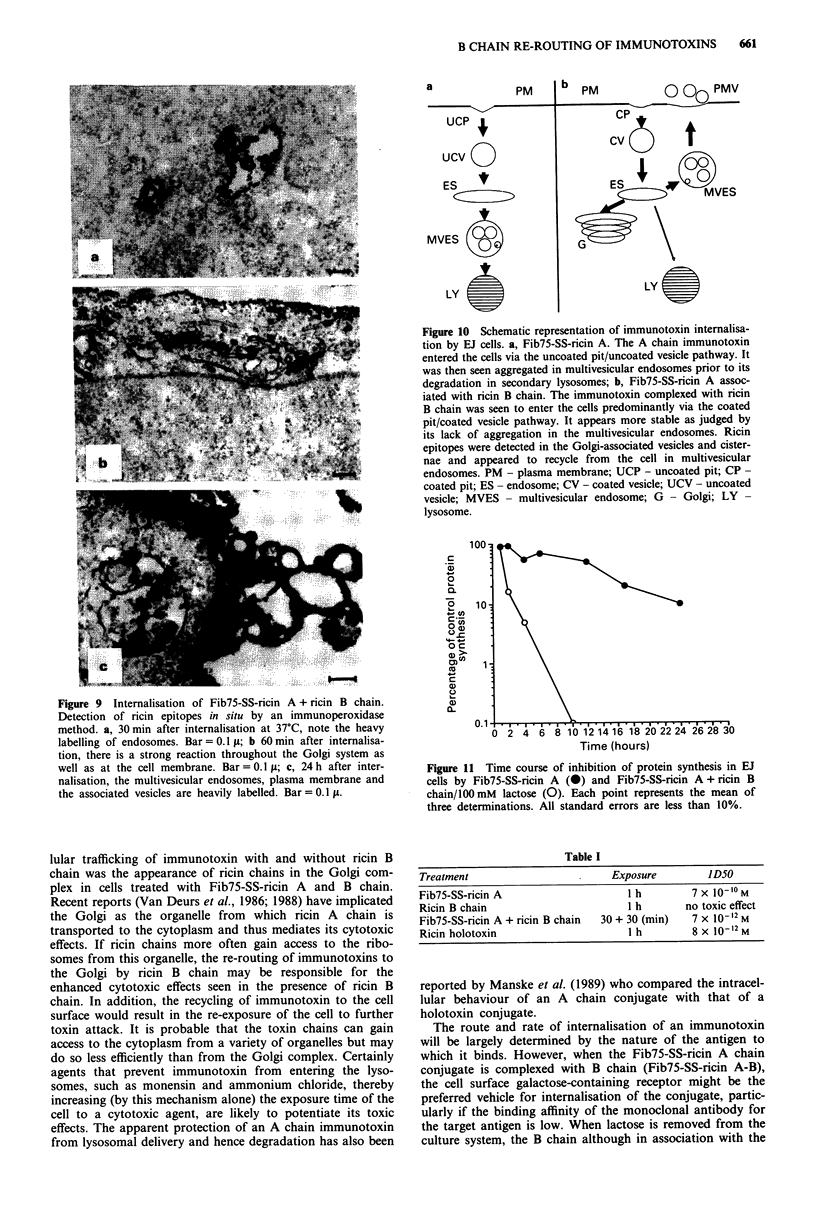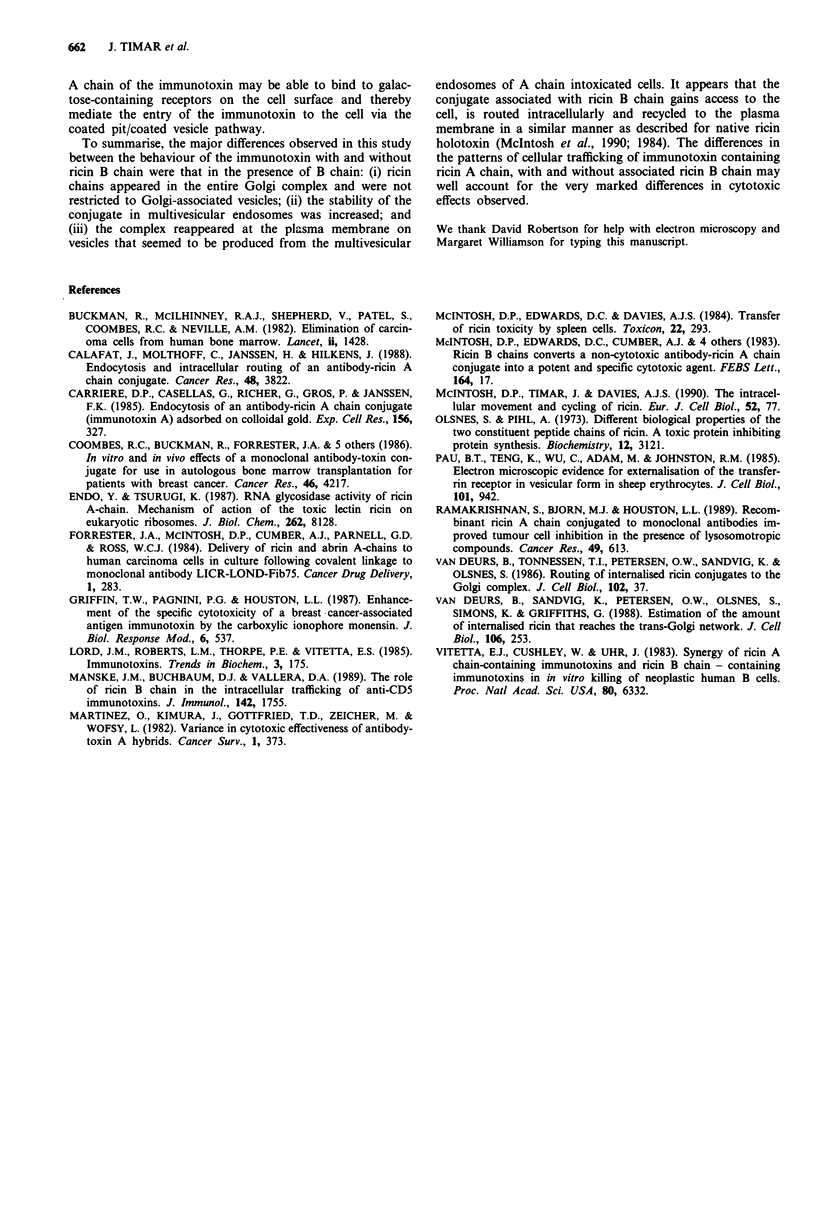# The effect of ricin B chain on the intracellular trafficking of an A chain immunotoxin.

**DOI:** 10.1038/bjc.1991.377

**Published:** 1991-10

**Authors:** J. Timar, D. P. McIntosh, R. Henry, A. J. Cumber, G. D. Parnell, A. J. Davies

**Affiliations:** Institute of Pathology and Experimental Cancer Research, Semmelweis Medical University, Budapest, Hungary.

## Abstract

**Images:**


*Br. J. Cancer* (1991), **64**, 655–662 © Macmillan Press Ltd., 1991

### The effect of ricin B chain on the intracellular trafficking of an A chain immunotoxin

J. Timar[1], D.P. McIntosh[2], R. Henry[3], A.J. Cumber[3], G.D. Parnell[3] & A.J.S. Davies[3]

[1]*Institute of Pathology and Experimental Cancer Research, Semmelweis Medical University, Budapest VIII, ULLOI UT 26, H-1085, Hungary;* [2]*Department of Pharmacy, University of Manchester, Manchester M13 9PL; and* [3]*Haddow Laboratories, Institute of Cancer Research, Sutton, Surrey SM2 5PX, UK.*

**Summary** Covalent linkage of the A chain of ricin to the LICR-LOND-Fib75 monoclonal antibody produced an immunotoxin, Fib75-SS-ricin A, which demonstrated immunospecific toxicity to human bladder carcinoma cells in tissue culture (Forrester *et al.*, 1984). The present studies have shown that ricin B chain potentiates the toxicity of the immunotoxin by two orders of magnitude and also significantly increases the rate of protein synthesis inhibition. Using immunoelectron microscopy, the receptor-mediated endocytosis and intracellular routing of the immunotoxin was studied with and without ricin B chain treatment after immunolocalisation of the conjugate. Fib75-SS-ricin A was internalised by the EJ cells predominantly in uncoated pits and vesicles and directed to the endosomes. Some degradation of the complex appeared to take place in multivesicular endosomes at early timepoints and 24 h after internalisation, most of the immunotoxin was found in lysosomes. Some ricin A chain epitopes were detected in Golgi vesicles. Cells treated with immunotoxin and ricin B chain endocytosed the complex predominantly in coated pits and coated vesicles. Using pre-embedding immunoperoxidase techniques, ricin chains were found in the whole Golgi complex and most of the conjugate escaped lysosomal degradation. Internalised immunotoxin was recycled back to the plasma membrane in an active form associated with vesicles which appeared to be derived predominantly from multivesicular endosomes. A similar mode of recycling has recently been reported (McIntosh *et al.*, 1990) for ricin holotoxin in the same cell line. These observations may explain the potentiating effect of toxin B chains in the antibody-directed targeting of toxin A chains.

The plant toxin ricin comprises two polypeptide subunits, A and B, joined by a disulphide bond. It binds to galactose-containing receptors on the surface of mammalian cells via its B chain (Olsnes & Pihl, 1973). The toxin subsequently enters the cells by receptor-mediated endocytosis where its toxicity is expressed by the inactivation of protein synthesis following the translocation of the A chain to the cytoplasm. Hybrid molecules, comprising monoclonal antibodies chemically coupled to the A chain of plant toxins (immunotoxins) (Lord *et al.*, 1985) have shown promise in the treatment of experimental animal tumours and are presently proving to be increasingly useful alternatives to conventional diffusible drugs in the treatment of neoplastic disease (Coombes *et al.*, 1986). Immunotoxins act by terminating protein synthesis in the target cells. Following binding by antibody recognition of plasma membrane antigens, conjugates enter cells by receptor-mediated endocytosis (Carriere *et al.*, 1985). After gaining access to the cytoplasm, the toxin A chain of the conjugate catalytically cleaves adenine residue 4324 in the 28S ribosomal RNA of the 60s ribosomal subunit, irreversibly halting cellular protein synthesis (Endo & Tsurugi, 1987). Thus the antibody molecule acts as a surrogate B chain to bring about immunospecific cell death. The LICR-LOND-Fib75 (Fib75) monoclonal antibody recognises an HLA class I-related plasma membrane antigen found in all differentiated human cells except those of the lymphoid system (Buckman *et al.*, 1982). Fib75 was covalently linked to the A chain of ricin to produce an immunotoxin that displayed an immunospecific and moderate toxicity to target cells in culture, but which was two orders of magnitude less toxic than the parent toxin (Forrester *et al.*, 1984). A-chain immunotoxins which lack the galactose-binding B chain polypeptides are often less cytotoxic than their holotoxin counterparts (Martinez *et al.*, 1982). Hence different methods have been employed to potentiate the cytotoxicity of A chain conjugates while maintaining their immunospecificity. Agents which have been investigated for their ability to potentiate immunotoxin activity include the lysosomotropic amines, ammonium chloride and chloroquine (Ramakrishnan *et al.*, 1989), carboxylic ionophores such as monensin (Griffin *et al.*, 1987) and the addition of free or antibody-conjugated toxin B chains following immunlocalisation of the A chain conjugate (Vitetta *et al.*, 1983; McIntosh *et al.*, 1983). The amines and ionophores are thought to increase immunotoxin potency by causing a rise in the pH of intracellular organelles thereby interfering with the passage of the immunotoxins from endosomes to lysosomes and also by inhibiting degradative enzyme activity. The aim of this study was to define and compare the mode of entry and subsequent routing of the native antibody-labelled Fib75-SS-ricin A chain conjugate in a human bladder carcinoma cell line with and without the presence of ricin B chain as a potentiating agent.

### Materials and methods

### *Tissue culture and cytotoxicity assays of target cells*

The EJ cell line is derived from a human bladder carcinoma and is routinely maintained in a 1:1 mixture of RPMI 1640/DMEM (Gibco BRL) supplemented with 5% foetal calf serum (FCS) (Gibco BRL) in a humidified atmosphere of 8% $CO_2$ in air at 37°C. For cytotoxicity studies, cells were plated out in Nunclon 24 well culture plates at $2 \times 10^5$ cells per well and incubated overnight to adhere to the plastic. Triplicate wells were exposed to log dilutions of conjugates, toxin A chains, antibodies or antibodies plus A chains for 1 h before washing the cells three times in PBS to remove unbound materials. For experiments in which B chain was used as a potentiating agent, after exposure to the conjugate, the cells were incubated with ricin B chain at $10^{-7}$ M for an additional hour in the presence of 100 mM lactose. The cultures were then incubated for a further 27 h before pulsing with $^3$H-leucine at 1 μCi per well for 17 h. At the end of this period, the cell monolayers were washed three times with PBS to remove the unincorporated isotope, fixed twice in 5% trichloracetic acid and dried following a methanol wash. The contents of each well were solubilised in 200 μl 1 M NaOH for 1 h at 37°C and protein synthesis was determined by scintillation counting 150 μl of cellular digest. The results are

Correspondence: D. McIntosh, Department of Pharmacy, University of Manchester, Oxford Road, Manchester M13 9PL, UK.
Received 5 September 1990; and in revised form 26 April 1991.

expressed as percentages of the levels of synthesis in appropriate control cultures. In experiments where the timecourse of protein synthesis inhibition was assayed, cells were exposed to either ricin, Fib75-SS-ricin A or conjugate plus ricin B chain for 1 h. For the earliest (1 h) timepoints, 5 μCi $^3$H-leucine of a higher specific activity than that used for the standard assays (120–190 Ci mMol$^{-1}$) was added to the cultures exposed to the treatments in leucine-free medium. For subsequent timepoints, the cells were washed free of toxin, conjugate and conjugate plus B chain/lactose and incubated in serum-free medium. Cultures were then pulsed for 20 min before harvesting as described above. For ultrastructural studies $4 \times 10^5$ cells were plated out (in triplicate) overnight in 30 mm Petri dishes (Sterilin) to allow the cells to adhere before processing. A mouse bladder carcinoma cell line MB49 was used as a specificity control.

*Antibodies*

Gold conjugates, anti-rabbit (G10) and anti-mouse (G5) IgGs were obtained from Janssen (Belgium). A polyclonal rabbit anti-ricin antibody was a gift from Dr D.C. Edwards (ICR, London, UK). It was raised against ricin toxoid and assayed for affinity for ricin holotoxin and A and B chains by an Ouchterlony assay. Fib75-SS-ricin A was prepared as described by Forrester et al. (1984). Briefly, the covalent linkage of ricin A chain to the Fib75 monoclonal antibody was achieved by first reacting the immunoglobulin with N-succinimidyl-3-(2-pyridyldithio) propionate (SPDP) under conditions that gave approximately two pyridylsulphide groups per molecule of IgG. The derivatised IgG was then allowed to react with a 2-fold molar excess of free A chain overnight at room temperature. The reaction mixture was resolved by gel filtration on Sephacryl S-200. Fractions corresponding to a 1:1 conjugate of antibody with A chain were used for this study. The conjugate was found to contain some free antibody as well as species singly and multiply substituted with toxin A chain.

*Prepartion of ricin B chain*

Essentially this method involves cleavage of the holotoxin disulphide bond while the toxin is immobilised on an agarose matrix via the binding site of the B chain. One hundred to 150 mg of the toxin was loaded onto an acid-treated Sepharose 4B column (bed volume 25 ml) equilibrated with 0.1 M phosphate buffer, pH 8.0, containing 1 mM EDTA. The column was then washed with four bed volumes of running buffer to elute both non and partially bound material. Running buffer (15 ml) containing 5% 2-mercaptoenthanol was loaded and the flow of buffer was then stopped and the column allowed to stand for 2 h to allow cleavage of the disulphide linkages. The toxin A chain was eluted leaving the B chain and uncleaved toxin still bound to the column. The B chain was eluted from the column with 500 mM galactose, 100 mM $Na_2PO_4$, 5% 2-mercaptoethanol, 150 mM NaCl pH 8.8. The eluted protein peak was dialysed against the running buffer for ion exchange chromatography. A column of LKB CM-Trisacryl (40 ml bed volume) was equilibrated with 10 mM phosphate buffer at pH 6.0 containing 100 mM galactose and 0.5% 2-mercaptoethanol and set to run at 14 ml h$^{-1}$ collecting 4 ml fractions. The dialysed B chain was applied to the column and the breakthrough peak containing the unbound fraction was collected.

The B chain was dialysed against the running buffer for chromatofocussing. A column of PBE94 (bed volume 30 ml) was equilibrated with 0.025 M histidine/HCl buffer at pH 6.2 containing 20 mM galactose and approximately 50 mg of dialysed B chain was applied to the column. The column was washed with 2–3 column volumes and a pH gradient formed by eluting with 12 column volumes of 1/8 dilution of Polybuffer 74 at pH 4.0 containing 20 mM galactose. The diluent used was distilled water and 1.0 M HCl to adjust the pH of the polybuffer. Pure B chain was focussed at its PI of 4.8.

*Ultrastructural immunocytochemistry*

The target cells were incubated with $10^{-7}$ M Fib75-SS-ricin A at 4°C for 15 min in medium containing FCS. The cells were then washed three times in cold phosphate buffered saline (PBS). In experiments in which ricin B chain was used following immunotoxin binding, $10^{-7}$ M ricin B chain was added to the Fib75-SS-ricin A-labelled cells in the presence of 100 mM lactose for an additional 15 min at 4°C. At the end of the incubation the cells were washed three times in PBS. The antibody component of the conjugate was labelled by incubation with a 1/10 dilution of a goat anti-mouse IgG-G5 conjugate (Janssen) for 15 min at 4°C. The cells were then washed three times in PBS to remove the unbound label. The A chain of the toxin was labelled by a 15 min incubation with a polyclonal rabbit anti-ricin antibody (22 μg ml$^{-1}$) followed by a 1/10 dilution of G10-labelled goat-anti-rabbit conjugate. To follow the fate of ricin B chain in the cells the B chain was bound to the cell surface at 4°C for 15 min at $10^{-7}$ M and the cells were washed as described above. The surface-bound ligand was then labelled with a rabbit anti-ricin polyclonal antibody (22 μg ml$^{-1}$). Labelling was completed with the addition of a goat anti-rabbit gold conjugate (G10). Preparations for the identification of plasma membrane bound ligands were fixed in 1% glutaraldehyde: 0.08 M cacodylate buffer pH 7.2 for 1 h at room temperature and processed as described below. Other cells were re-incubated in serum-containing medium at 37°C for various times up to 20 h to allow the internalisation of the labelled complex and then washed and fixed as described above.

*Processing of cells for electron microscopy*

After the first fixation, all cells were washed in cacodylate buffer and post fixed in 1% $OSO_4$ in the same buffer for a further hour. The monolayers were then washed with distilled water for 30 min and dehydrated in a graded series of alcohols. The cells were floated off the surface of the Petri dishes with propylene oxide. The cell pellet was centrifuged at 1,000 r.p.m. and embedded in Araldite (EMScope). Ultra thin sections were cut using a diamond knife, stained in lead citrate and examined in a Phillips EM-100 electron microscope at an accelerating voltage of 80 Kv.

*Quantification*

Ten randomly selected cells were photographed at a minimum magnification of 10,000 ×. The 10 nm gold particles in subcellular compartments labelling ricin chains were counted and expressed as a percentage of the total particle number at a given time point. The total particle number per cell at each time point exceeded 300.

*Binding and internalisation of native ligands*

Immunotoxin or B chain-treated immunotoxin was bound to the cell surface as described above and internalisation of ligands was allowed to continue for different time periods. The cells were washed in PBS and fixed in 2% paraformaldehyde/PBS for 30 min. The cells were then washed in PBS and permeabilised with 0.05% saponin/PBS (wt/v) for 45 min at room temperature after which the cells were washed extensively in PBS. Intracellular ricin A and B chains were detected by a 1 h incubation of the monolayer with rabbit-anti-ricin IgG (22 μg ml$^{-1}$) in PBS containing 0.05% saponin followed by extensive washes with PBS and a second antibody incubation with swine-anti-rabbit IgG-peroxidase conjugate (1/15 dilution in PBS/0.05% saponin). After three washes in PBS the cell monolayers were incubated for 30 min at room temperature in 2 ml PBS containing 1 mg diaminobenzidine (DAB) and 2 μl $H_2O_2$ (15%). After several PBS washes the cell monolayer was fixed in $OSO_4$ and processed for electron microscopy as described above. Fib75 monoclonal antibody, either free or conjugated, was detected by a 1 h incubation with a rabbit-anti-mouse IgG peroxidase conjugate at a 1:25 dilution in PBS/0.05% saponin. The cells were then processed as described previously. Preparations lacking the primary antibody served as negative controls for ricin A chain detection. For Fib75 controls, a rabbit-anti-goat IgG-peroxidase conjugate was used. Additionally, a mouse bladder carcinoma cell line, MB49 was used as specificity control.

### Results

### *Plasma membrane localisation of gold-labelled ligands at 4°C*

The immunotoxin was found in a patchy distribution on the plasma membrane appearing as a mixutre of 5 and 10 nM gold particles (monoclonal antibody and ricin A chain respectively) (Figure 1a, b). The Fib75-SS-ricin A with associated ricin B chain was found localised in coated pits as has been observed previously for ricin holotoxin (Van Deurs *et al.*, 1986; McIntosh *et al.*, 1990) as well as in discrete areas of the plasma membrane (Figure 2a). Fib75 antibody was seen as fairly evenly distributed label on the plasma membrane but was absent from coated areas. In the presence of 100 mM lactose no binding of ricin B chain to the cell surface was seen (data not shown). B chain added to cell monolayers without Fib75-SS-ricin A and in the absence of 100 mM lactose bound to the plasma membrane and was seen as small clusters of G10 gold label. No plasma membrane labelling was observed when either the MB49 mouse tumour was treated in place of the EJ cells or when the primary antibodies were omitted from the labelling procedures.

### *Internalisation of gold-labelled ligands*

After 5 min at 37°C the immunotoxin molecules were transferred to cytoplasmic endosomal compartments. The immunotoxin complex without B chain entered the cell exclusively in uncoated pits and vesicles (Figure 1c) while immunotoxin bearing ricin B chain entered cells predominantly (83%) in coated pits and coated vesicles, in a manner previously described for ricin holotoxin (Figure 2a and b). Components of Fib75-SS-ricin A were seen occasionally separated in early endocytic compartments suggesting a certain degree of lability of the conjugate.

In treatments using free B chain, the B chain was seen in the endosomes; some still remained on the plamsa membrane. Thirty to 60 min after internalisation of the immunotoxin complexes the most heavily labelled compartments were the endosomes and to a lesser extent the multivesicular endosomes; some label was also observed in secondary lysosomes (Figures 3a, b, c and 4a, b, c). All of these compartments contained both G5 and G10 gold particles indicating no further dissociation of the conjugate. In addition to the continuous presence of label at the plasma membrane, cells treated with Fib75-SS-ricin A plus ricin B chain started to generate vesicles labelled with both 5 nm and 10 nm gold particles which appeared at the cell surface; these vesicles were absent in cells treated with either Fib75-SS-ricin A alone or ricin B chain alone (McIntosh *et al.*, 1990). In cells internalising the immunotoxin-gold complex the gold particles were found to be aggregated in some multivesicular endosomes indicating that degradation of the protein component of the complex was taking place (Figure 3d). Aggregation of gold label in multivesicular endosomes at this timepoint was not seen in cells treated with immunotoxin plus ricin B chain and was not observed as a consequence of ricin toxicity in a previous study (Figure 4c). Twenty-four hours after internalisation of the complex the Fib75-SS-ricin A-treated cells contained the 5 and 10 nm gold complexes mostly in secondary lysosomes (Figure 5). Cells treated with the immunotoxin plus ricin B chain showed signs of cellular damage and contained far less lysosome-associated gold label (Figure 6b); most of the label was found extracellularly, mainly associated with plasma membrane vesicles (Figure 6a). In control samples both Fib75 antibody and ricin B chain were found in the lysosomes 24 h after internalisation and no cell surface labelling remained.

**Figure 1** Fib75-SS-ricin A immunolocalisation at 4°C on EJ cells. G5 gold label represents Fib75 and G10, ricin epitopes. **a**, label is localised in a well-defined area. Bar = 0.1 μ; **b**, Coated pit is unlabelled. Bar = 0.1 μ; **c**, Fib75-SS-ricin A, 5 min after incubation at 37°C. Note the presence of label at the cell surface and in uncoated pit/vesicles. There is an apparent separation of G5 and G10 particles in certain vesicles. There is an apparent separation of G5 and G10 particles in certain vesicles. Bar = 0.1 μ.

### *Quantification of gold label*

Morphometric analysis (based on calculations of 3,000 particles per sample) of the amount of G10 label (ricin A chain) found in cellular compartments showed significant differences between the internal distribution of Fib75-ricin A chain complexes with and without ricin B chain. It should be noted that ricin B chain may also have been labelled with the G10 since the polyclonal antibody used was raised against ricin holotoxin. The immunotoxin and B chain complex was found

**Figure 2** a, Localisation of Fib75-SS-ricin A associated with ricin B chain, at 4°C on EJ cells. Note the labelled membrane areas as well as the coated pit. Bar = 0.1 μ. b, Cells after incubation with conjugate plus B chain at 37°C for 5 min. The coated vesicle contains both the G5 and G10 label. Bar = 0.1 μ.

**Figure 3** Internalisation of Fib75-SS-ricin A at 37°C for 30 min. **a,b** Endosomes containing both G5 and G10 label. Bar = 0.1 μ. **c**, Multivesicular endosome labelled with G5 and G10. Bar = 0.1 μ. **d**, After 1 h at 37°C, Fib75-SS-ricin A in multivesicular endosomes in an aggregated form. Bar = 0.1μ.

**Figure 4** Internalisation of Fib75-SS-ricin A + B chain for 30 min. **a**, Labelled endosome. Bar = 0.1 μ. **b**, Labelled multivesicular endosome. Bar = 0.1 μ. **c**, Note lack of aggregated gold label in multivesicular endosome. Bar = 0.1 μ.

to enter the cells more slowly than the immunotoxin alone (Figure 7a). Once inside the cell both types of complex were found in acidic compartments but the levels found at 10–60 min after internalisation were greater for the immunotoxin alone than for the B chain-treated complex (Figure 7b). There was a significant difference between the amount of labelled Fib75-SS-ricin A and the immunotoxin with B chain found in the lysosomes at times later than 30 min after uptake by the cell (Figure 7c). Approximately 80% of the cell-associated Fib75-SS-ricin A was found in the lysosomes at 24 h compared with only 15% of the conjugate plus B chain.

*Internalisation of the native (unlabelled) ligands*

The preceding experiments clearly demonstrated differences in the mode of internalisation and fate of the two conjugates

**Figure 5** Fib75-SS-ricin A 24 h after internalisation by EJ cells at 37°C. Note the aggregated label in secondary lysosomes. Bar = 1.0 μ.

**Figure 6** Fib75-SS-ricin A + B chain 24 h after internalisation at 37°C. **a**, The plasma membrane and associated vesicles are heavily labelled with both G5 and G10. The cells infrastructure is altered due to the toxic effects of the conjugate. Bar = 1.0 μ. **b**, A secondary lysosome containing aggregated label. Bar = 1.0 μ.

(+ and − B chain). However it could be argued that their fates were influenced by the labelling method employed (gold particles). Therefore in the following experiments we have investigated the mode of internalisation of the unlabelled immunotoxins using peroxidase-labelled antibodies and permeabilised cells. The Fib75-SS-ricin A chain conjugate was seen to enter cells exclusively via uncoated pits and uncoated vesicles similarly to the gold-labelled conjugate. Later it was found in endosomes (Figure 8a). After 1 h of internalisation A chain was detected in Golgi-associated vesicles but none was detected in parallel membranes of the Golgi cisternae (Figure 8b). At later timepoints most of the immunotoxin was found in secondary lysosomes (Figure 8c). Fib75-SS-ricin A complexed with ricin B chain bound to the plasma membrane at 4°C in a manner similar to Fib75-SS-ricin A alone, but after warming to 37°C was seen to localise spontaneously in coated pits, enter the cells predominantly in coated vesicles and gain access to the endosomal system (Figure 9a). At timepoints later than 30 min, label was found in multivesicular endosomes (Figure 9c) and in the parallel membranes of the Golgi complex (Figure 9b). At later timepoints, label was found on extracellular vesicles associated with the plasma membrane in a manner identical to that seen with the gold-labelled Fib75-SS-ricin A + ricin B chain complex. Very little immunotoxin with B chain was found in the secondary lysosomes when the native ligands were stained with peroxidase. Figure 10 summarises our observations of the intracellular trafficking of Fib75-SS-ricin A (panel a) and Fib75-SS-ricin A treated post-immunolocalisation with ricin B chain (panel b). No labelling was observed when the anti-rabbit-goat IgG-peroxidase conjugate was substituted for the swine-anti-rabbit peroxidase conjugate or when the MB49 mouse cell line was given identical treatments in place of the EJ cell line.

*Cytotoxicity studies*

Table I shows the toxicity of 1 h exposures of the EJ cells to the ricin A chain conjugate with and without ricin B chain (in the presence of 100 mM lactose) as a second stage reagent. It can be seen that the ricin B chain treatment in conjunction with Fib75-SS-ricin A increased the toxicity of the conjugate by two orders of magnitude, resulting in a toxic effect similar to that of ricin holotoxin. A 1 h treatment of the cells with ricin B chain alone did not elicit an inhibitory response. Figure 11 compares the timecourse of protein synthesis inhibition in EJ cells by Fib75-SS-ricin A with and without ricin B chain treatment. A maximum of 90% inhibition was achieved 24 h after treatment with the conjugate alone. The t1/2 (time taken to inhibit protein synthesis by 50%) was 12.5 h. Treatment of cells with Fib75-SS-ricin A and ricin B chain resulted in total inhibition of protein synthesis and a t1/2 of 1.5 h, a figure comparable with that of ricin holotoxin (t1/2 = 1 h).

### Discussion

The present study has demonstrated that the moderate cytotoxic effects of Fib75-SS-ricin A can be enhanced significantly by a post-immunolocalisation treatment with ricin B chain. In addition, the rate of protein synthesis inhibition is markedly increased in the presence of the B chain. These considerations led us to speculate on the mode of internalisation of the Fib75-SS-ricin A conjugate in the absence and presence of ricin B chain and to determine whether there might be a correlation between the pattern of internalisation and the levels of toxicity seen in the target cells. It seems

**Figure 7** Quantitative analysis of the internalisation of ricin epitopes into EJ cells. (O) Fib75-SS-ricin A; ( + ) Fib75-SS-ricin A + ricin B chain. **a**, Label associated with the cell surface; **b**, Label associated with intracellular acidic compartments (endosomes and multivesicular endosomes); **c**, Lysosomal labelling (see Materials and methods).

**Figure 8** Internalisation of Fib75-SS-ricin A (for 1 h) by EJ cells. Detection of ricin epitopes by an indirect peroxidase method. **a**, The lumenal membrane of the endosome is clearly labelled; **b**, Golgi zone. Note the absence of ricin chains in the Golgi cisternae but the presence of label in Golgi-associated vesicles. Bar = 0.1 μ; **c** 24 h after internalisation of the conjugate. Ricin epitopes are found in secondary lysosomes. Bar = 0.1 μ.

likely that the endocytic route taken by the conjugate might play an important role in determining the intracellular organelle from which ricin A chain enters the cytoplasm and hence the kinetics of protein synthesis inhibition and the level of the resultant toxicity.

The present study of the internalisation of the native and gold-labelled conjugates following binding to the target cells at 4°C revealed that the intracellular trafficking of the two types was very similar. Both the native and gold-labelled conjugate bound to the plasma membrane and entered the cells predominantly by the uncoated vesicle pathway and were seen in the endosomal compartments, in multivesicular endosomes and at later times in secondary lysosomes. During the internalisation and routing of the conjugate both major components of the complex, epitopes of the monoclonal antibody and the ricin A chain, were found together in almost all intracellular compartments involved. A low level of segregation of the two target ligands was observed in some early endocytic compartments close to the plasma membrane. Only the native A chain of Fib75-SS-ricin A was seen to reach the Golgi-associated vesicles but not the Golgi cisternae. This may be due to the increased size of the immunotoxin-gold complex compared with the molecular size of the native immunotoxin. The presence of both sizes of gold particles in the majority of intracellular organelles indicates the stability of the conjugates disulphide linkage even in acidic compartments. Separate administration of Fib75 antibody and ricin B chain to the cells also resulted in the eventual passage of these ligands to the lysosomes. The routing of a monoclonal antibody and a ricin A chain immunotoxin to the lysosomes has also been reported by Calafat *et al.* (1988) who also observed that the immunotoxin remained intact until it reached the lysosomal compartment. In the present study although no apparent recycling of the conjugate to the cell surface was seen to occur in cells treated with Fib75-SS-ricin A, Fib75 antibody or ricin B chain alone, cells treated with immunotoxin plus B chain generated labelled vesicles at the cell surface (using both the gold and peroxidase labels) that seemed to derive from multivesicular endosomes. In a previous study of the intracellular trafficking of ricin holotoxin (McIntosh *et al.*, 1990) we have observed and measured ricin being returned to the cell surface associated with vesicles being released to the extracellular space from multivesicular endosomes. Subsequently this material was shown to be toxic in cytotoxicity assays (McIntosh, unpublished data). Exocytosis of vesicles from multivesicular endosomes during the cellular cycling of ligands is similar to that described for the release of the transferrin receptor by reticulocytes (Pau *et al.*, 1985).

The other major difference observed between the intracel-

**Figure 9** Internalisation of Fib75-SS-ricin A + ricin B chain. Detection of ricin epitopes *in situ* by an immunoperoxidase method. **a**, 30 min after internalisation at 37°C, note the heavy labelling of endosomes. Bar = 0.1 μ; **b** 60 min after internalisation, there is a strong reaction throughout the Golgi system as well as at the cell membrane. Bar = 0.1 μ; **c**, 24 h after internalisation, the multivesicular endosomes, plasma membrane and the associated vesicles are heavily labelled. Bar = 0.1 μ.

**Figure 10** Schematic representation of immunotoxin internalisation by EJ cells. **a**, Fib75-SS-ricin A. The A chain immunotoxin entered the cells via the uncoated pit/uncoated vesicle pathway. It was then seen aggregated in multivesicular endosomes prior to its degradation in secondary lysosomes; **b**, Fib75-SS-ricin A associated with ricin B chain. The immunotoxin complexed with ricin B chain was seen to enter the cells predominantly via the coated pit/coated vesicle pathway. It appears more stable as judged by its lack of aggregation in the multivesicular endosomes. Ricin epitopes were detected in the Golgi-associated vesicles and cisternae and appeared to recycle from the cell in multivesicular endosomes. PM – plasma membrane; UCP – uncoated pit; CP – coated pit; ES – endosome; CV – coated vesicle; UCV – uncoated vesicle; MVES – multivesicular endosome; G – Golgi; LY – lysosome.

**Figure 11** Time course of inhibition of protein synthesis in EJ cells by Fib75-SS-ricin A (●) and Fib75-SS-ricin A + ricin B chain/100 mM lactose (○). Each point represents the mean of three determinations. All standard errors are less than 10%.

lular trafficking of immunotoxin with and without ricin B chain was the appearance of ricin chains in the Golgi complex in cells treated with Fib75-SS-ricin A and B chain. Recent reports (Van Deurs *et al.*, 1986; 1988) have implicated the Golgi as the organelle from which ricin A chain is transported to the cytoplasm and thus mediates its cytotoxic effects. If ricin chains more often gain access to the ribosomes from this organelle, the re-routing of immunotoxins to the Golgi by ricin B chain may be responsible for the enhanced cytotoxic effects seen in the presence of ricin B chain. In addition, the recycling of immunotoxin to the cell surface would result in the re-exposure of the cell to further toxin attack. It is probable that the toxin chains can gain access to the cytoplasm from a variety of organelles but may do so less efficiently than from the Golgi complex. Certainly agents that prevent immunotoxin from entering the lysosomes, such as monensin and ammonium chloride, thereby increasing (by this mechanism alone) the exposure time of the cell to a cytotoxic agent, are likely to potentiate its toxic effects. The apparent protection of an A chain immunotoxin from lysosomal delivery and hence degradation has also been reported by Manske *et al.* (1989) who compared the intracellular behaviour of an A chain conjugate with that of a holotoxin conjugate.

**Table I**

| *Treatment* | *Exposure* | *ID50* |
|---|---|---|
| Fib75-SS-ricin A | 1 h | $7 \times 10^{-10}$ M |
| Ricin B chain | 1 h | no toxic effect |
| Fib75-SS-ricin A + ricin B chain | 30 + 30 (min) | $7 \times 10^{-12}$ M |
| Ricin holotoxin | 1 h | $8 \times 10^{-12}$ M |

The route and rate of internalisation of an immunotoxin will be largely determined by the nature of the antigen to which it binds. However, when the Fib75-SS-ricin A chain conjugate is complexed with B chain (Fib75-SS-ricin A-B), the cell surface galactose-containing receptor might be the preferred vehicle for internalisation of the conjugate, particularly if the binding affinity of the monoclonal antibody for the target antigen is low. When lactose is removed from the culture system, the B chain although in association with the

A chain of the immunotoxin may be able to bind to galactose-containing receptors on the cell surface and thereby mediate the entry of the immunotoxin to the cell via the coated pit/coated vesicle pathway.

To summarise, the major differences observed in this study between the behaviour of the immunotoxin with and without ricin B chain were that in the presence of B chain: (i) ricin chains appeared in the entire Golgi complex and were not restricted to Golgi-associated vesicles; (ii) the stability of the conjugate in multivesicular endosomes was increased; and (iii) the complex reappeared at the plasma membrane on vesicles that seemed to be produced from the multivesicular endosomes of A chain intoxicated cells. It appears that the conjugate associated with ricin B chain gains access to the cell, is routed intracellularly and recycled to the plasma membrane in a similar manner as described for native ricin holotoxin (McIntosh *et al.*, 1990; 1984). The differences in the patterns of cellular trafficking of immunotoxin containing ricin A chain, with and without associated ricin B chain may well account for the very marked differences in cytotoxic effects observed.

We thank David Robertson for help with electron microscopy and Margaret Williamson for typing this manuscript.

### References

BUCKMAN, R., MCILHINNEY, R.A.J., SHEPHERD, V., PATEL, S., COOMBES, R.C. & NEVILLE, A.M. (1982). Elimination of carcinoma cells from human bone marrow. *Lancet*, **ii**, 1428.

CALAFAT, J., MOLTHOFF, C., JANSSEN, H. & HILKENS, J. (1988). Endocytosis and intracellular routing of an antibody-ricin A chain conjugate. *Cancer Res.*, **48**, 3822.

CARRIERE, D.P., CASELLAS, G., RICHER, G., GROS, P. & JANSSEN, F.K. (1985). Endocytosis of an antibody-ricin A chain conjugate (immunotoxin A) adsorbed on colloidal gold. *Exp. Cell Res.*, **156**, 327.

COOMBES, R.C., BUCKMAN, R., FORRESTER, J.A. & 5 others (1986). *In vitro* and *in vivo* effects of a monoclonal antibody-toxin conjugate for use in autologous bone marrow transplantation for patients with breast cancer. *Cancer Res.*, **46**, 4217.

ENDO, Y. & TSURUGI, K. (1987). RNA glycosidase activity of ricin A-chain. Mechanism of action of the toxic lectin ricin on eukaryotic ribosomes. *J. Biol. Chem.*, **262**, 8128.

FORRESTER, J.A., MCINTOSH, D.P., CUMBER, A.J., PARNELL, G.D. & ROSS, W.C.J. (1984). Delivery of ricin and abrin A-chains to human carcinoma cells in culture following covalent linkage to monoclonal antibody LICR-LOND-Fib75. *Cancer Drug Delivery*, **1**, 283.

GRIFFIN, T.W., PAGNINI, P.G. & HOUSTON, L.L. (1987). Enhancement of the specific cytotoxicity of a breast cancer-associated antigen immunotoxin by the carboxylic ionophore monensin. *J. Biol. Response Mod.*, **6**, 537.

LORD, J.M., ROBERTS, L.M., THORPE, P.E. & VITETTA, E.S. (1985). Immunotoxins. *Trends in Biochem.*, **3**, 175.

MANSKE, J.M., BUCHBAUM, D.J. & VALLERA, D.A. (1989). The role of ricin B chain in the intracellular trafficking of anti-CD5 immunotoxins. *J. Immunol.*, **142**, 1755.

MARTINEZ, O., KIMURA, J., GOTTFRIED, T.D., ZEICHER, M. & WOFSY, L. (1982). Variance in cytotoxic effectiveness of antibody-toxin A hybrids. *Cancer Surv.*, **1**, 373.

MCINTOSH, D.P., EDWARDS, D.C. & DAVIES, A.J.S. (1984). Transfer of ricin toxicity by spleen cells. *Toxicon*, **22**, 293.

McINTOSH, D.P., EDWARDS, D.C., CUMBER, A.J. & 4 others (1983). Ricin B chains converts a non-cytotoxic antibody-ricin A chain conjugate into a potent and specific cytotoxic agent. *FEBS Lett.*, **164**, 17.

MCINTOSH, D.P., TIMAR, J. & DAVIES, A.J.S. (1990). The intracellular movement and cycling of ricin. *Eur. J. Cell Biol.*, **52**, 77.

OLSNES, S. & PIHL, A. (1973). Different biological properties of the two constituent peptide chains of ricin. A toxic protein inhibiting protein synthesis. *Biochemistry*, **12**, 3121.

PAU, B.T., TENG, K., WU, C., ADAM, M. & JOHNSTON, R.M. (1985). Electron microscopic evidence for externalisation of the transferrin receptor in vesicular form in sheep erythrocytes. *J. Cell Biol.*, **101**, 942.

RAMAKRISHNAN, S., BJORN, M.J. & HOUSTON, L.L. (1989). Recombinant ricin A chain conjugated to monoclonal antibodies improved tumour cell inhibition in the presence of lysosomotropic compounds. *Cancer Res.*, **49**, 613.

VAN DEURS, B., TONNESSEN, T.I., PETERSEN, O.W., SANDVIG, K. & OLSNES, S. (1986). Routing of internalised ricin conjugates to the Golgi complex. *J. Cell Biol.*, **102**, 37.

VAN DEURS, B., SANDVIG, K., PETERSEN, O.W., OLSNES, S., SIMONS, K. & GRIFFITHS, G. (1988). Estimation of the amount of internalised ricin that reaches the trans-Golgi network. *J. Cell Biol.*, **106**, 253.

VITETTA, E.J., CUSHLEY, W. & UHR, J. (1983). Synergy of ricin A chain-containing immunotoxins and ricin B chain – containing immunotoxins in *in vitro* killing of neoplastic human B cells. *Proc. Natl Acad. Sci. USA*, **80**, 6332.